# Patient-reported outcome measures (PROMs) to personalise follow-up care of ovarian cancer: what do patients think? A qualitative interview study

**DOI:** 10.1007/s00520-024-08436-z

**Published:** 2024-03-26

**Authors:** Dachel D. Seeratan, Robin G. van Schuylenburch, Luc R. C. W. van Lonkhuijzen, Johanna W. M. Aarts

**Affiliations:** 1 Department of Obstetrics and Gynaecology, Amsterdam UMC, Location University of Amsterdam, Meibergdreef 9, Amsterdam, the Netherlands; 2 Department of Gynaecological Oncology, Cancer Centre Amsterdam, Amsterdam, the Netherlands

**Keywords:** Patient-reported outcome measures (PROMs), Ovarian cancer, Qualitative interview, Quality of life, Follow-up care

## Abstract

**Purpose:**

The purpose of this study was to explore ovarian cancer patients’ preferences regarding follow-up care and, in particular, the use of patient-reported outcome measures (PROMs) as an approach to personalise follow-up care.

**Methods:**

Between May and June 2021, semi-structured interviews were conducted with ovarian cancer patients, who had finished their primary treatment at least 6 months prior and were receiving follow-up care at our centre. Interviews were transcribed verbatim and analysed using an inductive thematic approach. A thematic flow chart was created describing interacting themes.

**Results:**

Seventeen patients were interviewed, of which 11 were familiar with PROMs. Two key themes emerged from the data: the need for reassurance and the wish for personalised care. A follow-up scheme using PROMs was identified as a separate theme with the potential to personalise care. Several barriers and facilitators of PROMs were mentioned.

**Conclusions:**

Ovarian cancer patients have a desire for personalised follow-up care and seek reassurance. PROMs may be able to support both of these needs. Future research is needed to determine the most effective, patient-centred way to implement them.

**Implications for cancer survivors:**

By understanding what patients’ preferences are regarding follow-up care, more initiatives can be set up to personalise follow-up care, through which patient anxiety and dissatisfaction can be reduced.

**Supplementary Information:**

The online version contains supplementary material available at 10.1007/s00520-024-08436-z.

## Introduction

Ovarian cancer is one of the top ten most commonly occurring cancers in women, with a 5-year survival rate of 37.6% [1]. After a primary treatment, patients enter a routine 5-year follow-up period. Within those 5 years, around 70% of patients have a recurrence of disease [2]. When the disease recurs, treatment will always be palliative. Living with the fear of recurrence is a daily struggle for ovarian cancer patients and has a large impact on their quality of life (QoL) and their family’s well-being [3, 4].

Treatment is often long and intense with early and late side effects leading to an impaired QoL. In addition, women with ovarian cancer often do not feel sufficiently involved in their follow-up care and have unmet needs [5]. These needs, as well as the impact of treatment on their QoL, vary among patients. Therefore, there is a growing interest in personalising follow-up care. The use of patient-reported outcome measures (PROMs) has been investigated as a means to personalise care for multiple types of cancers [6, 7]. PROMs are tools or instruments to measure the patient’s self-perceived health status, such as health-related quality of life [8]. There are some important advantages to the use of PROMs in care: (1) clinicians can pay specific attention to patients’ reported needs in the limited time available at consult [9], (2) patients can prepare for their appointments by self-reflection and (3) barriers for bringing up topics that are difficult to discuss are reduced for both patients and clinicians [10]. The use of PROMs as part of a type of home monitoring system has shown potential benefits for health care organisations [7].

Within the field of gynaecological oncology PROMs have been used in clinical practice. Several studies showed that PROMs can play a beneficial role in the personalisation of follow-up care for these women [11–13]. Patient acceptance is found to be strong and a wide-range of benefits for patients have been shown, e.g. the ability to gain new insights in their problems.

To date, no study has examined ovarian cancer patients’ preferences for the use of PROMs, whether or not as a part of a remote monitoring system. The objective of this study is to explore women’s preferences for follow-up care, and, in particular, the use of PROMs as an approach to personalising follow-up care.

## Methods

### Design

We performed a qualitative study with semi-structured interviews. The medical ethics committee of the Amsterdam University Medical Centre (METC) approved this study on May 27th 2021 (referral number: W21_253 # 21.278app) in accordance with the Declaration of Helsinki.

### Setting

This single-centre study was conducted in the Amsterdam University Medical centres between May and June 2021. All gynaecological cancer patients visiting the outpatient clinic are requested to complete a PROMs questionnaire prior to their appointment as part of routine clinical care. Completion occurs online through the electronic patient portal. Initially, a paper-based version was offered, which was sent to patients’ home addresses before their consultation. However, with the paper version, patients’ answers could not easily be retrieved and compared during future outpatient consultations. Therefore, the department of gynaecological oncology decided to only provide the PROMs questionnaire digitally. Currently, 60% of gynaecological cancer patients at our centre fill out the PROMs. The PROMs include questions from the validated QoL questionnaires (QLQ) C30, QLQ-CX-24, QLQ EN-24, QLQ OV-28 and QLQ-VU34 from the European Organisation for Research and Treatment of Cancer (EORTC) [14]. The questionnaire consists of 80 questions and includes questions specifically aimed at patients with ovarian cancer.

### Participants

Patients were eligible if they were above the age of 18, finished their primary treatment at least 6 months prior to the interview period, received their follow-up care at our medical centre and were able to communicate in Dutch. Although patients are asked to complete PROMs before their outpatient clinic visit as part of routine care, it was not a requirement for participation in the interviews. We screened upcoming outpatient schedules for eligible patients. Before approaching patients for participation, we requested permission from their treating physician. Written informed consent was obtained before the start of the interview.

### Interview guide

An interview guide was developed based on clinical expertise and existing literature. The following topics were included: For each topic, an open-ended question with corresponding probes was formulated. The interview guide was piloted within the research group and with one patient, after which it underwent revisions. The final version of the interview guide is enclosed in the Supplementary Information section.

### Data collection

Participants were invited for a one-time interview led by one of the researchers (RS). By using a semi-structured approach based on the interview guide, a two-way dialogue was stimulated. All interviews were conducted in Dutch and audio recorded.

All interviews were transcribed verbatim, and all data were anonymized using generic descriptions, for example Amsterdam would be replaced by ‘city’. Interviews continued until data saturation was reached, which was confirmed with two additional interviews. Background and clinical characteristics of the participants were collected from the electronic patient record.

### Data analysis

Transcripts were analysed using the inductive thematic approach developed by Braun & Clarke [15, 16] and the practical guide of Saunders et al. [17]. We worked ‘bottom-up,’ which means the analysis was not guided by a known theoretical framework.

The data transcripts were independently coded line-by-line by two researchers (RS and DS) using the program MAXQDA 2022, after which coding discrepancies were discussed. Any remaining discrepancies were resolved with a senior author (JA). Codes were grouped into themes, after which all transcripts were read again to ensure that the themes captured all of the material. A thematic map was created to depict associations and interactions between themes and codes. Both coders and the senior author agreed on the final thematic map. For mapping and creating flow charts, the visual collaboration platform Miro was used [18]. For the purpose of this paper, quotes were selected and translated to English using DeepL [19] and checked for content by two authors (DS and RS).

## Results

Forty-four outpatient clinic appointments were scheduled between May 10th and June 11th 2021, of which 24 patients were eligible for this study based on our inclusion criteria. Four patients were deemed ineligible by their treating physician, for example based on limited proficiency in Dutch. Nineteen patients were contacted via telephone by one of the researchers (RS), of which two declined participation stating a lack of time or interest. Seventeen women were included. Interviews lasted around 30 to 50 min and took place following an appointment at the outpatient clinic. One of the participants was interviewed while admitted in the hospital for administration of a cycle of palliative chemotherapy for recurrent disease.

### Patient characteristics

The median age was 69 years (range 25–81). The median duration that patients were in follow-up was 48 months (range 3–168). Patients included in this study had various stages and types of ovarian cancer, and therefore also received different types of treatment regimes (Table 1). Eight patients had recurrent disease. As part of usual follow-up care, seven patients regularly completed PROMs prior to their appointment. Six completed PROMs once or twice during the follow-up period, but not on a regular basis. Four had never completed PROMs and/or were not aware of their existence.

**Table 1:** Patient characteristics

Patient	Age	FIGO stage	Type of ovarian carcinoma	Type of treatment	End of initial treatment	Duration follow-up (months)^a^	Recurrence of disease	Time until first recurrence (months)^b^	Filled out PROMs (yes/no)	Distance to hospital (km)
1	63	III-B	High-grade serous	NACT-IDB + HIPEC-ACT-PARP	Jan-21	3	No	NA	Yes	73
2	72	IV-B	High-grade serous (papillar)	NACT-IDB-ACT	Dec-19	16	No	NA	Once	< 15
3	63	IV-A	High-grade serous	NACT-IDB + HIPEC-ACT	Nov-20	6	No	NA	Yes	< 15
4	39	IV-B	Clear cell	DB-ACT	Jun-19	23	Yes	5	Once	79
5	81	III-B	Pathology unknown	NACT-IDB	Nov-20	6	No	NA	Yes	50
6	74	III-C	High-grade serous	DB-ACT-PARP	Jan-20	16	No	NA	Once	Abroad
7	60	III-B	Low-grade serous	DB-ACT	Aug-20	9	No	NA	Yes	22
8	69	II-C	High-grade serous	DB-ACT	Nov-10	126	Yes	12	No	70
9	75	I-C	Low-grade serous (papillar)	DB-ACT	Jan-06	168	Yes	144	Once	< 15
10	52	I-C	Low-grade mucinous	Staging laparotomy	Feb-17	50	No	NA	No	< 15
11	73	III-C	High-grade serous	Chemotherapy	Nov-19	24	Yes	9	Once	< 15
12	25	I	Serous borderline tumour	Laparoscopic unilateral cystectomy + unilateral adnexectomy	Nov-12	108	Yes	13	Yes	< 15
13	63	I	Granulosacell	AUE + unilateral adnexectomy	Sep-2016	60	No	NA	No	< 15
14	71	I-C3	High-grade serous	Unilateral adnexectomy + staging laparotomy + chemotherapy	Oct-2015	72	Yes	24	Yes	29
15	46	IV-A	Adenocarcinoma, NOS	NACT-IDB-ACT	Oct-2017	48	Yes	31	Once	21
16	81	III-C	High-grade serous	DB-ACT	May-2010	72	Yes	36	No	24
17	74	I-A	Granulosacell	AUE + BSO	Jun-14	84	No	NA	Yes	20

*ACT *adjuvant chemotherapy, *AUE *abdominal uterus extirpation, *BSO *bilateral salpingo oophorectomy, *DB *debulking surgery, *HIPEC *hyperthermic intraperitoneal chemotherapy, *IDB *interval debulking surgery, *NACT *neoadjuvant chemotherapy, *PARP *poly (adp-ribose) polymerase inhibitors, *NA *not applicable, *NOS *not otherwise specified

^a^Duration of follow-up is defined as the time between the end of the initial treatment until the time of the interview

^b^Time until first recurrence is defined as the time between the end of the initial treatment, and the first confirmation (based on imaging, pathology or lab results) of recurrence of disease

### Defining themes

**Fig. 1:**
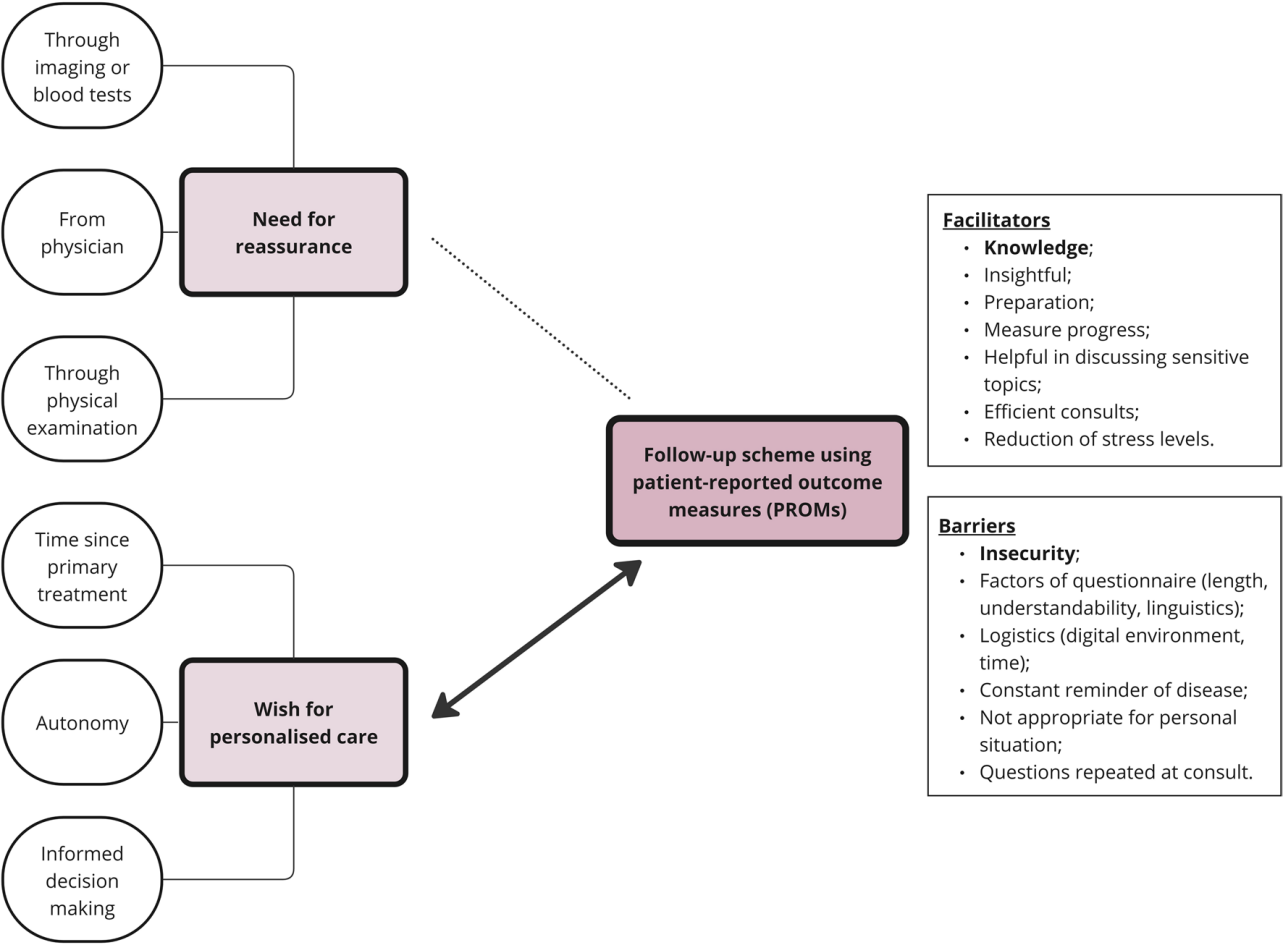
Flow chart of thematic analysis. Dotted line indicates possible interaction according to patients. Thick arrowed line indicates very likely interaction based on patients’ response

Two key themes regarding follow-up care were developed based on the data: (1) the need for reassurance and (2) the wish for personalised care. PROMs itself was crafted as a third key theme about which patients expressed possible barriers and facilitators in the (regular) use as part of their follow-up care. We found that PROMs can most likely facilitate the preference for personalization of care, and possibly the need for reassurance. An explanatory model of these findings was developed in the form of a flow chart (Fig. 1).

### Need for reassurance

All patients stated that having ovarian cancer made them very insecure, mainly as they did not experience significant physical symptoms before diagnosis. Based on this insecurity, all 17 patients said seeking reassurance was their main goal for follow-up appointments. Seven patients mainly sought verbal reassurance from the physician that they were doing well. Four patients sought reassurance through blood tests (e.g. tumour markers). They stated that the feeling of insecurity strongly depended on the level of the tumour marker. Other patients mentioned their need for imaging for the same reason. They stated that they could never know what was going on inside their bodies without imaging.

And hence also the blood test, with the value. If I see that the value is eight, then I just know like: Guys it’s—I’m stable.” And that’s just the most important thing.—Patient 7

Nine patients thought it was very important that physical examination (PE) took place at each appointment. They believed that the gynaecologist could have a better idea of what was going on inside their bodies, when he/she completed a PE. Those patients could not feel reassured about the state of their cancer without the PE.

(…) But if you don’t get checked, you have to hope, knock on wood, that you don’t get any symptoms. Then you would have to call again and then you don’t know what to expect. So I just like those check-ups. – Patient 13

It gives you a little bit of confidence that she’s [gynaecologist] not feeling weird things, because apparently she can feel if it’s not as smooth and she feels bumps and things like that. Should something [cancer] come back.—Patient 14

Four patients did not deem PE necessary at each consultation. Two of them believed its necessity depended on imaging results or the presence of symptoms. One patient expressed that she trusted the physician to decide when PE was necessary. One patient struggled to comprehend the reduction in PE frequency over time after ending primary treatment.

At first, it was difficult for me, but more in the sense of understanding how does she [gynaecologist] feel a difference from three months ago when she sees 20 women every day?—Patient 14

Insecurity emerged as a common theme for all patients, driving their need for reassurance. Patients described various forms of insecurity, such as losing trust in their bodies due to the absence of clear symptoms during diagnosis. Some said they lived in constant fear of recurrence, influencing the plans they made in their lives, while others made the conscious decision to only worry when a recurrence should occur.

(…) because during the first few years you’re still afraid. You still have that fear of whether it [cancer] will come back or not. Actually not only during the first few years; you still have it. I don’t think it [the fear] will ever go away. It will stay, but after such a check-up you are reassured.—Patient 9

### Wish for personalised care

We identified three factors interacting with the key theme defined as the wish for personalisation of care: time since primary treatment, autonomy and informed decision-making. Patients reported that their needs and wishes changed as more time passed since primary treatment. Their needs did not necessarily align with the suggested follow-up schemes. Nevertheless, quite a few patients indicated that they preferred to rely on the recommendation of the physician to decide the frequency of their appointments.

Patients voiced the use of their autonomy in certain situations, such as when deciding whether or not physical examination should take place. This mostly depended on whether physical symptoms were present or not. Patients voiced a similar opinion with regard to imaging and blood tests. Some patients mentioned that regular testing of tumour markers made them more insecure, as the result was often higher than reference values even without signs of recurrence.

(…) but with no physical complaints and a good result from the CT scan, I don’t think physical examination is necessary.—Patient 13

On the contrary, multiple patients did not fully understand the reasons why certain choices in follow-up trajectories were made: they experienced a lack of knowledge and/or a lack of transparent communication by the clinicians. For example, some patients did not understand why routine testing of tumour markers and imaging was not done.

(…) And it is actually the case that the women who did get an MRI scan and blood tests, were more depressed. And because they want to keep quality of life as optimal as possible, they’re not going to do that [routine testing or imaging]. So that just applies nationwide. But I do think that it’s personal-it’s different for everybody. I would be more depressed from the fact that I don’t know anything, than if I have clarity.—Patient 6

Quite a few patients wondered if they would have enough knowledge to make personal choices about their follow-up plan, hampering their informed decision-making. However, all patients expressed personal preferences for the ideal frequency and manner of follow-up care, for example via telephone or in-patient consultations. Even though the majority was content with the current frequency of appointments, a few patients thought the ideal frequency depended on the physician’s opinion.

Especially because I do trust her [gynaecologist] in that [deciding on follow-up frequency]. If she says: “I don't think it’s wrong.” Then it’s [reducing follow-up frequency] fine.—Patient 11

Some patients expressed a wish for more remote follow-up care via telephone, especially when they are not experiencing symptoms. Nonetheless, the majority preferred in-patient visits, because of their need for reassurance.

Well first of all, I think you can also have a good conversation over the phone. I noticed that for example in [country], because it was really in the middle of that—It was the second wave of the pandemic there, so I wasn’t allowed to come in. But I had to be checked. (…) Well then I opted for the phone call. Afterwards I thought, “well I don’t actually have any complaints, so it’s not bad at all. “But that call did matter.—Patient 2

### Follow-up scheme using patient-related outcome measures (PROMs)

In terms of willingness to fill out PROMs regularly, five patients agreed to do so as long as their answers were utilised during consultations. However, six patients encountered too many barriers and preferred not to fill them out. Two patients were willing to fill out PROMs but not prior to each consultation, suggesting an annual or biannual frequency.

The majority of patients felt that filling out PROMs was for the physician’s benefit in preparing consultations and gaining insights into patients’ conditions.

But I do understand that it’s kind of nice for a doctor to know how I’m doing, because it’s a lot of questions as you’ve seen, so [pause] but generally those kinds of questions just get asked during the consultation.—Patient 14

One patient thought PROMs could help in alerting supporting out-of-hospital specialists, such as home care or psychologists, when necessary.

During the interviews, the interviewer described a scenario to all patients as to how PROMs can be used in follow-up care as a type of home monitoring system (see Supplementary Information for the full interview guide):

Suppose you complete the questionnaires at home on the computer and send them in; and depending on your answers, you will be invited to your appointment at the hospital or not. How would you feel about that?—Interviewer

The majority stated they would not prefer this personalised follow-up scheme based on PROMs. Two patients found the proposed method agreeable to implement straight away, and an additional two might find it agreeable in the future. Patients saw potential benefits of the addition of PROMs to regular appointments, but were not ready to replace in-person consultations.

Of course you can cheat on paper and say: “nothing, nothing, nothing” [referring to symptoms or complaints]. But I could still look very pale and have no radiance. That should be a sign for the doctor to know that something is wrong.—Patient 5

Patients expressed openness to a potential personalised home monitoring system based on PROMs, but they emphasised the importance of clear instructions on how to deal with symptoms or problems and when to contact healthcare providers. Besides that, they would like to be contacted if the physicians think that anything alarming came up in their responses. A few patients expressed concerns about potential waiting times for physical consultations when they would have to book these themselves when symptoms occur, which would hinder their willingness to call healthcare providers themselves.

### Barriers and facilitators

All patients had encountered at least some barriers when completing the PROMs questionnaires. Most patients found the questionnaire was too long, and others (*n* = 4) struggled with the clarity and interpretation of questions, particularly those with Likert-scale answer options. For some patients, differentiating symptoms from other comorbidities or accurately assessing symptom severity was challenging.

Another barrier was that questions were often perceived as intrusive when they were not relevant to the patients’ personal situation, such as inquiries about sexuality after the loss of a spouse or questions that felt inappropriate due to their age. Therefore, they felt they should have the freedom to decide themselves whether or not they wanted to complete certain parts of the questionnaire, depending on whether they felt the question related to their current situation.

No, the only thing is that sometimes they then ask in those questionnaires: “how much are you restricted with grocery shopping or everyday things?” […] that doesn’t apply to me at all as a 25-year-old. But I can imagine someone in their 70’s or 80’s having trouble with those things. So not all questions are entirely appropriate for my age group as well.—Patient 12

Multiple patients considered filling out PROMs redundant, as they were asked similar questions during their appointment.

Well I thought it was meant as preparation, so that the doctor can read it and then uh—[silence]. But she asks half of the questions again, and then especially those questions of which I think: “you have this [the answers] in black and white.” Whereas those other questions that you can’t ask so easily in black and white, she doesn’t ask those either.—Patient 2

Patients considered taking up a more prominent role in their follow-up schemes, which led to a more positive attitude towards using PROMs. Patients emphasised the importance of understanding the relevance and purpose of PROMs, as this knowledge facilitated their willingness to regularly complete them. The current understanding of its relevance and purpose was widespread. Some patients believed that PROMs primarily benefited the healthcare system by reducing in-person consultations, cutting costs, saving time and resources and making consultations more efficient. A few patients recognised the benefits for themselves, such as gaining insights into their bodies and diseases, monitoring their progress towards recovery, accessing supporting specialties more easily, reducing travel time and minimising hospital visits.

The less often you have to go to the hospital, the better that is and, so you can go on with your life. I think that’s always better than constantly facing your illness anyway.—Patient 13

I think the questionnaire in itself is a very good idea, because as I was saying; these are things I don't think about. It makes you more conscious after all. And yes. Sometimes you don't want to notice things about yourself. And this maybe is an eye-opener.—Patient 9

Some patients highlighted that using PROMs could facilitate discussions about sensitive topics, while others felt that this did not apply to them personally.

But I could imagine that eh… that you can put a topic on the schedule at that text box that you can still fill in. That you suggest something, geez maybe you want to talk about:… And that you can tick off a subject. (…) So that you don’t have to say that yourself. (…) It’s so hard to bring something like that to the table.—Patient 2

## Discussion

We discovered that, to ovarian cancer patients, there are two key aspects of follow-up care: (1) the need for reassurance and (2) the wish for personalised care. Patients expressed the wish for personalised follow-up care tailored to their specific needs, which appears to be highly dependent on the time since their primary treatment and their knowledge to make certain choices. Patients expressed that there is a great deal of insecurity associated with the disease and prognosis, which results in a need for reassurance. It seems that PROMs can contribute to personalising follow-up care. However, to patients, it did not seem to be self-evident without thorough information and knowledge. Patients need to be informed about the purpose and relevance of PROMs, as well as when and how to contact healthcare providers when problems occur. Most patients recognised benefits of filling out PROMs on a regular basis for physicians, the healthcare system and patients, such as gaining new insights in their bodies and disease and keeping track of their progress towards full recovery. Nevertheless, to fully implement PROMs in follow-up care successfully, some barriers need to be overcome and patients’ input is pivotal.

A prior study, the PROMova-study, explored ovarian cancer patients’ thoughts on the use of PROMs during consultations [20]. They found that, overall, patients found it useful to complete PROMs before a follow-up appointment. However, in this study, they did not consider the function of PROMs as an alternative to regular follow-up. Some of the patients we interviewed seemed to be open to using PROMs instead of an in-person visit, depending on several factors, such as being able to request a clinical appointment at any time. This is in line with the findings of the trial of Riis et al. (2020), in which personalised care with the use of electronic patient-reported outcomes (ePROs) for breast cancer patients, was compared with standard care without ePROs [7].

Patients in our study expressed feeling insecure, partially caused by lack of knowledge about what to expect during follow-up. In addition to their wish for more personalization of care, we argue that a higher level of shared decision-making (SDM) between patient and clinician is needed when discussing follow-up care plans. This includes the provision of tailored information, supported with patient information aids. It is known that this can reduce anxiety [21]. Moreover, in these SDM conversations, it is important to elicit what is important to the patient. PROMs could be an important facilitator in SDM [22].

Even though it is known that regular measurement of tumour markers or physical examination in asymptomatic ovarian cancer patients does not lead to greater survival [23–25], both seemed to be of great significance to the patients in our study. This was mostly caused by the need for reassurance from follow-up appointments. Kargo et al. (2019) proposed that pre-scheduled follow-up visits for asymptomatic patients can cause false reassurance [12]. In their review, they concluded that PROMs can help patients identify otherwise undetected symptoms and therefore might also fuel this need for reassurance, so that they do not feel they will miss early signs of recurrence. This is in line with patients’ speculations in this study.

Finally, it is of great importance to collaborate with patients when setting up and implementing an alternative follow-up care plan for them. We identified multiple facilitators and barriers that can influence future implementation of PROMs in care, which are mostly similar to those set out in the systematic review of Glenwright et al. [26]. In their review, they report an important barrier, which was not identified in our study: completing PROMs can be difficult for patients with low language or computer literacy, as well as for patients with physical or cognitive impairments. Future studies should assess how to make PROMs more inclusive to those patient groups.

### Strengths and limitations

Although some protocols are published of trials set up to evaluate the use of PROMs in follow-up care in ovarian cancer, to the best of our knowledge, this is the first study to evaluate ovarian cancer patients’ preferences on follow-up care and the implementation of PROMs. Given the qualitative nature of this study, an in-depth review of topics could be achieved providing a real sense of how patients feel about these topics. There are some limitations to this study. Our population was selected using convenience sampling from a single tertiary cancer centre, which resulted in a population that consists of patients diagnosed with mostly advanced FIGO stage disease. This might not reflect the preferences of patients with early stage of ovarian cancer. Furthermore, preferences regarding the use of PROMs in follow-up care are partly based on hypothetical situations portrayed by the interviewer. During the time of interviews, the COVID-19 pandemic was still ongoing in The Netherlands, during which remote monitoring was promoted by the hospital and government. As such. the patients that were interviewed had been more exposed to remote monitoring as compared to other years. Another factor that makes this study less generalizable, is that patients were only exposed to the PROMs questionnaire that was used at our centre. We cannot exclude that a certain culture exists in our clinic, in which the use of PROMS is promoted. Patients’ opinions might be influenced by this, although interviewees did not hesitate to express concerns or be critical about the concept.

## Conclusion

Participants of this study expressed a strong need for reassurance through follow-up care and a desire for more personalised care. The use of PROMs in follow-up care might be an important method to tailor care to the specific needs of the individual patient. However, it needs to be implemented well. The findings of this study can aid in the design of future studies or implementation efforts. More research is needed to determine the manner of implementation that will be most effective and beneficial, e.g. in addition to regular follow-up or as a type of home monitoring system.

### Supplementary Information

Below is the link to the electronic supplementary material.

Supplementary file1 (DOCX 18 KB)

Supplementary file2 (DOCX 16 KB)

## Data Availability

No datasets were generated during the current study. Parts of the transcripts can be made available from the corresponding author on reasonable request.

## References

[1] De Angelis R, Sant M, Coleman MP, Francisci S, Baili P, Pierannunzio D (2014). Cancer survival in Europe 1999–2007 by country and age: results of EUROCARE-5—a population-based study. Lancet Oncol.

[2] Ovarian Cancer Recurrence and Treatment | OCRA. OCRA. https://ocrahope.org/patients/diagnosis-and-treatment/recurrence/#:~:text=Patients%20diagnosed%20in%20Stage%201,95%20percent%20chance%20of%20recurrence. Accessed 8 Oct 2023

[3] Roberts K, Clarke CL (2009). Future disorientation following gynaecological cancer: women’s conceptualisation of risk after a life threatening illness. Health Risk Soc.

[4] Tan JH, Sharpe L, Russell HC (2020). The impact of ovarian cancer on individuals and their caregivers: a qualitative analysis. Psycho-oncology.

[5] Beesley VL, Price MA, Webb PM, O’Rourke P, Marquart L, Butow P (2012). Changes in supportive care needs after first-line treatment for ovarian cancer: identifying care priorities and risk factors for future unmet needs. Psycho-oncology.

[6] Denis F, Lethrosne C, Pourel N, Molinier O, Pointreau Y, Domont J et al (2017) Randomized trial comparing a web-mediated follow-up with routine surveillance in lung cancer patients. J Natl Cancer Inst 109(9). 10.1093/jnci/djx029 10.1093/jnci/djx029 28423407

[7] Riis CL, Jensen PE, Bechmann T, Möller S, Coulter A, Steffensen KD (2020). Satisfaction with care and adherence to treatment when using patient reported outcomes to individualize follow-up care for women with early breast cancer—a pilot randomized controlled trial. Acta Oncologica.

[8] Weldring T, Smith SM (2013). Article commentary: patient-reported outcomes (PROs) and patient-reported outcome measures (PROMs). Health Serv Insights.

[9] Greenhalgh J, Gooding K, Gibbons E, Dalkin S, Wright J, Valderas JM et al (2018) How do patient-reported outcome measures (PROMs) support clinician-patient communication and patient care? A realist synthesis. J Patient Rep Outcomes 2(1). 10.1186/s41687-018-0061-6 10.1186/s41687-018-0061-6 PMC6153194 30294712

[10] Lavallee DC, Chenok KE, Love R, Petersen C, Holve E, Segal C (2016). Incorporating patient-reported outcomes into health care to engage patients and enhance care. Health Affairs.

[11] Nama V, Nordin A, Bryant A (2013) Patient-reported outcome measures for follow-up after gynaecological cancer treatment. Cochrane Libr 2022(1). 10.1002/14651858.cd010299.pub2 10.1002/14651858.CD010299.pub2 PMC6457831 24249483

[12] Kargo AS, Coulter A, Jensen PE, Steffensen KD (2019) Proactive use of PROMs in ovarian cancer survivors: a systematic review. J Ovarian Res 12(1). 10.1186/s13048-019-0538-9 10.1186/s13048-019-0538-9 PMC6631969 31307510

[13] Kennedy F, Shearsmith L, Holmes M, Rogers Z, Carter R, Hofmann U et al (2022) Electronic patient-reported monitoring of symptoms during follow-up of ovarian cancer patients: a feasibility study. BMC Cancer 22(1). 10.1186/s12885-022-09817-5 10.1186/s12885-022-09817-5 PMC9250717 35780095

[14] Aaronson NK, Ahmedzai SH, Bergman B, Bullinger M, Cull A, Duez N (1993). The European Organization for Research and Treatment of Cancer QLQ-C30: a quality-of-life instrument for use in international clinical trials in oncology. J Natl Cancer Inst.

[15] Braun V, Clarke V (2006). Using thematic analysis in psychology. Qual Res Psychol.

[16] Braun V, Clarke V, Rance N (2015) How to use thematic analysis with interview data. In: SAGE Publications Ltd eBooks, pp 183–97. 10.4135/9781473909847.n13

[17] Saunders CH, Sierpe A, Von Plessen C, Kennedy AM, Leviton LC, Bernstein SL (2023). Practical thematic analysis: a guide for multidisciplinary health services research teams engaging in qualitative analysis. BMJ.

[18] The visual collaboration platform for every team | Miro. https://miro.com/. Accessed 08-03-2024

[19] DeepL Translator (n.d.) Retrieved June 8, 2023, from https://www.DeepL.com/translator. Accessed 08-03-2024

[20] Kargo AS, Jensen PE, Lindemann K, Hjollund NH, Lund B, Haee M (2021). The PROMova study comparing active and passive use of patient-reported outcome measures in ovarian cancer follow-up: effect on patient-perceived involvement, satisfaction with care, and usefulness. Acta Oncol.

[21] Stacey D, Lewis KB, Barry MJ, Bennett C, Eden K, Holmes-Rovner M et al (2017) Decision aids for people facing health treatment or screening decisions. Cochrane Libr 2017(4). 10.1002/14651858.cd001431.pub5 10.1002/14651858.CD001431.pub5 PMC6478132 28402085

[22] Damman OC, Jani A, De Jong BA, Becker A, Metz MJ, De Bruijne MC (2020). The use of PROMs and shared decision-making in medical encounters with patients: an opportunity to deliver value-based health care to patients. J Eval Clin Pract.

[23] Rustin GJS, Van Der Burg MEL, Griffin C, Guthrie D, Lamont A, Jayson GC (2010). Early versus delayed treatment of relapsed ovarian cancer (MRC OV05/EORTC 55955): a randomised trial. Lancet.

[24] Clarke T, Galaal K, Bryant A, Naik R (2014) Evaluation of follow-up strategies for patients with epithelial ovarian cancer following completion of primary treatment. Cochrane Libr 2018(9). 10.1002/14651858.cd006119.pub3 10.1002/14651858.CD006119.pub3 PMC6457804 25198378

[25] Menczer J, Chetrit A, Sadetzki S, Golan A, Levy T (2006). Follow-up of ovarian and primary peritoneal carcinoma: the value of physical examination in patients with pretreatment elevated CA125 levels. Gynecol Oncol.

[26] Glenwright BG, Simmich J, Cottrell M, O’Leary SP, Sullivan C, Pole JD et al (2023) Facilitators and barriers to implementing electronic patient-reported outcome and experience measures in a health care setting: a systematic review. J Patient Rep Outcomes 7(1). 10.1186/s41687-023-00554-2 10.1186/s41687-023-00554-2 PMC9928985 36786914

