# Supplementary material for: Patient-reported outcome measures (PROMs) to personalise follow-up care of ovarian cancer: what do patients think? A qualitative interview study
Source: Support Care Cancer. 2024 Mar 26;32(4):247. doi: 10.1007/s00520-024-08436-z (PMC10963503; doi:10.1007/s00520-024-08436-z)
Supplement: Supplementary file 1 — Supplementary file1 (DOCX 18 KB) [file 520_2024_8436_MOESM1_ESM.docx]

**Original interview guide in Dutch**

*Wij kijken hoe we de nazorg voor patiënten met eierstokkanker kunnen verbeteren. In dit onderzoek willen we graag inzicht krijgen in de ervaringen die u heeft met het gebruiken van vragenlijsten in uw nazorg, en wat u zou vinden van een gepersonaliseerd nazorgschema op basis van deze vragenlijsten.*

*Het interview duurt ongeveer 30 minuten. Om de resultaten goed te kunnen analyseren zouden we het interview graag opnemen. Vindt u dat goed? [zo ja, dan dit antwoord ook nog opnemen op het bandje]*

Wilt u eerst kort wat vertellen uw behandel traject tot nu toe?

Kunt u mij vertellen hoe uw nazorg nu verloopt?

o   Hoe **vaak** heeft u een afspraak?

o   Met **wie** heeft u een afspraak?

o   **Wat** bespreekt u tijdens een afspraak?

Als u tussen uw afspraken in klachten bemerkt, wat doet u dan?

o   **Wacht** u op uw volgende afspraak?

o   Kijkt u het even aan? Zo ja, **hoelang**? (dagen/weken)

o   Weet u met wie u contact op moet nemen?

Wat is voor u het belangrijkste **‘’doel’’** van de controle afspraken?

Hoe heeft u de zorg het afgelopen jaar met de **COVID**-maatregelen ervaren?

o   Had u **minder** afspraken?

o   Verliepen uw afspraken **anders**; video-consult of telefonisch?

o   Zo ja, hoe beviel dit?

Hoe is het voor u naar het ziekenhuis te gaan voor controle afspraken, en wat maakt het **wel / niet** prettig?

o   Heeft uw **reistijd, reiskosten** hier invloed op?

o   Gaat er **iemand met u mee**; is dit lastig om te regelen?

o   Ervaart u **spanning** voor een afspraak, zo ja hoelang van tevoren?

o   Hoe voelt u zich na een controle afspraak?

o   Wat zou u ervan vinden als u **minder ziekenhuisbezoeken** heeft?

Wat vindt u van het lichamelijke onderzoek tijdens een controle afspraak?

o   Vindt er lichamelijk onderzoek plaats bij **elke afspraak**?

o   Ziet u **er tegen op**?

o   Is het voor uzelf **belangrijk** dat lichamelijk onderzoek plaatsvindt?

Krijgt u de vragenlijsten toegestuurd voor uw controle afspraken en zo ja vult u die in?

o   Vult u ze in op **papier of online (Mychart)**, waarom op die manier?

o   Welke **barrières** ervaart u bij het invullen?

o   Hoeveel **tijd** besteedt u aan het invullen?

o   Wat vindt u van de **hoeveelheid** vragen?

Worden de vragenlijsten gebruikt tijdens uw afspraken, en hoe?

Waarom denkt u dat wij u vragen om de vragenlijst in te vullen?

Zijn er onderwerpen onderbelicht volgens u in de vragenlijsten?

o   Zijn er vragen waarmee u **moeite** heeft? (bijv. vragen over seksualiteit/gemeenschap)

o   Zijn er vragen die u **overbodig/raar** vindt?

o   Zijn de vragen **relevant** voor u?

o   Begrijpt u waarom de vragen/onderwerpen **inhoudelijk** worden gesteld?

Helpt het u om **inzicht** te krijgen in hoe uw zich voelt en of u klachten heeft?

o   Vindt u het **nuttig**?

o   Hoe is het voor u om de vragenlijsten in te vullen?

o   Vindt u het **confronterend** om de vragenlijsten thuis in te vullen?

*Stel dat u thuis de vragenlijsten invult op de computer en op stuurt; afhankelijk van de ingevulde antwoorden wordt u wel of niet uitgenodigd voor uw afspraak in het ziekenhuis.*

Wat zou u daarvan vinden?

o   En als dat betekent dat u bijv. **een jaar niet fysiek** op controle hoeft te komen?

o   Wat zou u **missen** aan de fysieke controle afspraken?

o   Maakt het voor u uit **hoe lang** u al onder controle bent, 1,2,3,4 of 5 jaar?

Is het voor u een extra **drempel** om zelf om een afspraak aan te vragen?

*U vult de vragenlijsten nu om de 3 tot 6 maanden voorafgaand aan uw afspraak, als de afspraken worden aangepast op basis van ingevulde vragenlijsten, hoe vaak wilt u dan vragenlijsten invullen?*

**Vragenlijst invullen**; net zo vaak als nu of vaker? (Bijv. elke maand, 2 of 3 maanden)

Wat voor **terugkoppeling** zou u willen krijgen na het invullen?

o   Bij goed/ slecht nieuws

o   Telefonisch/Mail

o   Standaard/Persoonlijk bericht

o   Arts/ Poli-medewerker/ Vpk specialist

Wat vindt u er van als er op basis van uw ingevulde vragenlijst laagdrempelig afspraken gemaakt/aangeboden worden met **ondersteunende specialismen** (VPK, diëtiek, psycholoog, maatschappelijk werker?)

Zou dit (deze manier van zorg) uw **mening** over het invullen van vragenlijsten veranderen?

Kijkt u na dit gesprek anders naar hoe de follow-up is georganiseerd?

Hoe ziet voor u de ideale nazorg er uit?

o   Hebben de wensen van uw naasten hier invloed op?

**Heeft u nog vragen of opmerkingen voor mij?**
