# Supplementary material for: Patient-reported outcome measures (PROMs) to personalise follow-up care of ovarian cancer: what do patients think? A qualitative interview study
Source: Support Care Cancer. 2024 Mar 26;32(4):247. doi: 10.1007/s00520-024-08436-z (PMC10963503; doi:10.1007/s00520-024-08436-z)
Supplement: Supplementary file 2 — Supplementary file2 (DOCX 16 KB) [file 520_2024_8436_MOESM2_ESM.docx]

**Translated interview guide in English**

*We are looking at how we can improve follow-up care for patients with ovarian cancer. In this study, we would like to gain insight into your experiences with using questionnaires during follow-up care, and how you would feel about a personalised follow-up care plan based on these questionnaires.*

*The interview will last about 30 minutes. In order to properly analyse the results, we would like to record the interview. Do you agree? [if yes, please also record this answer on the tape].*

Will you first briefly tell me about your treatment process so far?

Can you tell me about your follow-up care at this time?

o   How **often** do you have an appointment?

o   With **whom** do you have an appointment?

o   **What** do you discuss during an appointment?

If you notice symptoms between your appointments, what do you do?

o   Do you **wait** for your next appointment?

o   Do you give it some time? If so, **how long**? (days/weeks)

o   Do you know who to contact?

What is the main **''purpose''** of the check-up appointments for you?

How did you experience care over the past year with **COVID** measures?

o  Did you have **fewer** appointments?

o   Did your appointments occur **differently**; video consultation or by phone?

o   If so, how did you feel about this?

What is it like for you to go to the hospital for check-up appointments, and what makes it **pleasant/difficult**?

o Does your **travel time, travel expenses** affect this?

o   Does someone **accompany** you; is this difficult to arrange?

o  Do you experience **anxiety** before an appointment, if so how long in advance?

o  How do you feel after a check-up appointment?

o  How would you feel about having **fewer hospital visits**?

How do you feel about the **physical examination** during a check-up appointment?

o Does physical examination take place at **each appointment**?

o Do you **dread it**?

o Is it **important** to you that physical examinations take place?

Do you receive the questionnaires for your check-up appointments and if so do you fill them out?

o Do you complete them on **paper or online** (Mychart), and why in that way?

o Which **barriers** do you experience when completing them?

o How much **time** do you spend completing them?

o How do you feel about the **amount** of questions?

Are the questionnaires used during your appointments, and how?

Why do you think we are asking you to complete the questionnaire?

Are any topics in the questionnaires underemphasized according to you?

o   Do you have **difficulty** with any questions? (e.g., questions about sexuality/community)

o   Do you find any questions **redundant/weird**?

o   Are the questions **relevant** to you?

o   Do you understand **substantively** why the questions/topics are addressed?

Does it help you to gain **insight** in how you feel and if you have symptoms?

o   Do you find it **helpful**?

o   What is it like for you to complete the questionnaires?

o   Is it **confronting** for you to complete the questionnaires at home?

*Suppose you complete the questionnaires at home on the computer and send them in; and depending on your answers, you will be invited to your appointment at the hospital or not.*

How would you feel about that?

o   And what if that means that you don't have to come for physical check-ups **for a year** for instance?

o   What would you **miss** about the physical check-up appointments?

o   Does it matter to you **how long** you have been under care; 1,2,3,4 or 5 years?

Is it an additional **barrier** for you to request an appointment yourself?

*You now complete questionnaires every 3 to 6 months prior to your appointment. If appointments are adjusted based on completed questionnaires, how often would you like to complete questionnaires?*

**Completing questionnaires**; as frequently as you do now or more often? (E.g. every month, 2 or 3 months)

What kind of **feedback** would you like to receive after completion?

o   In case of good/bad news

o Via phone/e-mail

o Standardized/Personal message

o Physician/clinical assistant/nurse practitioner

how would you feel if appointments with **supporting specialties** (nurse, dietician, psychologist, social worker?) were made lowkey based on your completed questionnaire?

Would this (this method of care) change your **opinion** about completing questionnaires?

Do you look differently at the structure of follow-up after this conversation?

What does the ideal follow-up care look like to you?

o   Do the wishes of your loved ones influence this?

**Do you have any questions or comments for me?**
